# A client-centered approach in home care for older persons – an integrative review

**DOI:** 10.1080/02813432.2020.1841517

**Published:** 2020-11-17

**Authors:** Päivi Sanerma, Sari Miettinen, Eija Paavilainen, Päivi Åstedt-Kurki

**Affiliations:** a Department of Health Sciences, Faculty of Social sciences, University of Tampere, Tampere, Finland; b Hamk Smart Reseach Unit, Hamk University of Applied Sciences, Hämeenlinna, Finland; c Information Services Unit, Research Unit, The Social Insurance Institution of Finland, Helsinki, Finland

**Keywords:** Client-centered care, older person, home care, home services, evidence-based nursing

## Abstract

**Objective:**

To describe and synthesize client-centered care and service in home care for older persons.

**Methods:**

The study was an integrative review using the guidelines for literature reviews by the Joanna Briggs Institute. The research process followed the Whittemore and Knafl framework and PRISMA toolkit in the selection of eligible articles. The CINAHL, Medline, Scopus, Web of Science and Social Sciences abstracts were searched for articles published between January 2007 and May 2020 according to previously designed search strategies. In total, 24 articles were deemed relevant for an analysis using a thematic analysis.

**Results:**

The analysis resulted in four themes with sub-themes which revealed that client-centered care and service in home care consist of: 1) Clients’ involvement in their own care; self-care, decision-making, satisfactory daily life, 2) Family members’ and care partners’ participation in care; family members’ and care partners’ commitment to care, family members’ and care partners’ competence in care, 3) Communication and co-operation; communication models, empowerment, partnership, and 4) Evidence-based service competence; delivery and organization of services, implementation of services, versatile clinical skills, quality outcomes and personnel wellbeing.

**Conclusions:**

According to the results, achieving client-centered care and service in home care requires the realization of all of the above aspects. The practice of nursing must better identify all dimensions of client-centered care and take these into account in the delivery of home care services.

**KEY POINTS**
- Client-centeredness is a fundamental value and the basis of nursing and care in home care provided for older persons
- This paper:
- deepens and structures the concept of client-centered care in the context of home care.
- assists professionals to understand the factors behind client-centered care within the home care environment.
- provides deeper understanding of the roles of the older person, family members, and the service system in developing client-centered services in home care for older persons.

## Introduction

In the European Union, older persons expect to receive increasingly high quality integrated home care services [1]. The home care service structure is influenced by state service structures, regulations, financial aspects, and available workforce. The key pillars of the home care of the future will be seamless integration and coordination of services, high quality, utilization of technology and client-centered care [2–5].

Client-centered care has been defined as ‘an approach to practice established through the formation and fostering of therapeutic relationships between all care providers, patients and others significant to them in their lives. It is underpinned by values of respect for persons, individual right to self-determination, mutual respect and understanding’ [6,7]. Client-centered care is focused on care needs, involvement, autonomy and respect. It is an approach to plan, deliver, and evaluate health care that relies on a mutually beneficial partnership, is well-planned and implemented, and is measured and evaluated in interprofessional collaboration where the client has an up-to-date care and service plan [6,8–12]. When clients experience a high quality of care and service, at the same time, the client-centeredness of service tends to be on a high level [13]. Concepts related to client-centered care include person-centered care and patient-centered care. Similarities can also be found in the concepts of user or customer-oriented services [10,12].

From the value base and principles of nursing, home care is guided by the ethics of care, self-determination, continuity of care and family-centeredness. A positive client–nurse relationship benefits seniors in two ways: they feel both comfortable and safe in the relationship to which they are committed. Research results highlight the importance of client–nurse interpersonal interactions and communication, which promote comfort and connectedness [14–17].

In the client-centered care process, the goals of care are negotiated in cooperation with the client's family members [4]. Coordination of services and nursing, the competence of professionals and economical organization of work are important factors influencing client-centeredness and quality. The level of collaboration with family members appears to be directly proportional to the quality of care and services [18–20]. This research topic is important because client-centered care is an ethical and fundamental value of home care. It increases a high quality of care and involvement of clients and families and increases the well-being of personnel [13].

The aim of the current integrative literature review is to describe and synthesize client-centered care and service in the home care of older people. The review is guided by the following question: How is client-centered care defined with respect to the older person’s home care and what factors underlying client-centered care have been identified in earlier studies?

## Methods

An integrative review is a method that allows the inclusion of diverse methodologies to provide a broad understanding about a particular phenomenon of interest. This integrative review followed the guidelines of the Joanna Briggs institute for a literature review. The Preferred Reporting Items for Systematic Review and Meta-Analysis (PRISMA) was utilized in the selection of eligible articles [21,22]. Quality assessment was performed using the JBI Critical Appraisal instruments depending on the study design [23] (Tables 1–4).

**Table 1.** Selected quantitative studies.

Researcher(s) and title of the article	Country
Brazil K, Bainbridge D, Ploeg J, Kruegel P, Marshall D. 2012. Family caregiver view on patient-centred care at the end of life. Scandinavian Journal of Caring science.	Canada
Bölenius K, Lämås K, Sandman PO, Edvardsson D. 2017. Effects and meanings of a person-centred and health-promoting intervention in home care services – a study protocol of a non-randomised controlled trial. BMC Geriatrics.	Sweden
Bosman R, Bours G, Engels J, Witte L. 2008. Client-centred care perceived by clients of the two Dutch homecare agencies: a questionnaire survey. International Journal of Nursing studies.	Netherlands
Sundler A, Höglander J, Håkansson J, Holmström I. 2017. Older person’s expressions of emotional cues and concern during home care visits. Application of the Verona codin definitions of emotional sequences (VR-CoDES) in home care. Patient Education and counseling.	Sweden
Höglander J, Håkansson J, Hilde E, Holmström I, Sundler A. 2017. Registered nurses’ and nurse assistants’ responses to older persons’ experssions of emotional needs in home care. Journal of Advanced nursing.	Sweden
Van Eenoo L, Roest H, Onder G, Finne-Soveri H, Garms-Homolova V, Jonsson P, Draisma S, Hout H, Declercq A. 2018. Organizational home care models across Europe: a cross sectional study. International Journal of Nursing studies.	Belgium
Parsons J, Parsons M. 2012. The effect of a designated tool on person-centered goal identification and service planning among older people receiving home care in New Zealand. Health and Social care in the Community.	New Zealand
Hafskjold L, Sundler A, Holmström I, Sundling V, Dulmen S, Hilde E. 2015. A cross-sectional study on person-centred communication in the care of older people: the COMHOME study protocol. BMJ open.	Norway
Turjamaa R, Hartikainen S, Kangasniemi M, Pietilä AM. 2015. Is it time for comprehensive approach in older home care client’s care planning in Finland? Scandinavian Journal of caring Sciences.	Finland
Hafskjold L, Sundling V, Eide H. 2018. Nursing staff’s responses to thematic content of patients’ expressed worries: observing communication in home care visits. BMC Health Services Research.	Norway

**Table 2.** Selected qualitative studies.

Researcher(s) and title of the article	Country
Roin A. 2017. Person-centredness in elder care: a secondary analysis of data from a study among home-dwelling men and women in Faroe Islands. Journal of Clinical nursing.	Faroe Islands
Kristensen D, Sundler A, Hafskjold L, Ruud I, Holmström I. 2017. Characteristics of communication with older people in home care: a qualitative analysis of audio records of home care visits. Journal of Clinical Nursing.	Norway
Öreland S, Määttä S, Norderg A, Winther Jörgensen M, Lutzen K. 2008. Nurses as guests or professionals in home health care. Nursing Ethics.	Sweden
Kuluski K, Peckham A, Gill A, Gagnon D, Wong-Cornall C, McKillop A, Parsons J, Sheridan N. 2019. What is important to older people with multimorbidity and their caregivers? Identifying attributes of person centered care from the user perspective. International Journal of Integrated Care.	Canada
Sundler A, Hjertberg F, Keri H, Holmström I. 2019. Attributes of person‐centred communication: a qualitative exploration of communication with older persons in home health care. International Journal of Older People Nursing.	Sweden

**Table 3.** Selected literature reviews.

Researcher(s) and title of the article	Country
Wilberforce M, Challis D, Davies L, Kelly M, Roberts C, Loynes N. 2016. Person-centredness in the care of older adults: a systematic review of Questionnaire-based scales and their measurement properties. BMC Geriatrics.	UK
Ruggiano N, Edvardsson D. 2013. Person-centeredness in home- and community-based long-term care: current challenges and new directions. Social work in Health Care.	USA
DePuccio M, Hoff T. 2014. Medical home interventions and quality outcomes for older adults: a systematic review. Quality Management in Health Care.	USA
Anker-Hansen C, Skovdahl K, McCormack B. 2018. The third person in the room: the needs of care partners of older people in home care services – a systematic review from a person-centred perspective. Journal of clinical nursing.	Norway
Carvajal A, Haraldsdottir T, Kroll T, McCormack B, Errasti-Ibarrondo B, Larkin P. 2019. Barriers and facilitators perceived by registered nurses to providing person-centred care at the end of life. A scoping review. International Practice Development Journal.	Spain
Giosa J, Holyoke P, Stolee P. 2019. Let’s get real about person- and family-centred geriatric home care: a realist synthesis. Canadian Journal on Aging.	Canada
Olsen C, Bergland A, Debesay J, Bye A, Langaas A. 2018. Striking a balance: health care providers’ experiences with home based, patient-centered care for older people – a meta-synthesis of qualitative studies. Patient Education and counseling.	Norway

**Table 4.** Selected case studies.

Researcher(s) and title of the article	Country
Silver G, Keefer J, Rosenfeld P. 2011. Assisting patients to age in place: an innovative pilot program utilizing the Patient Centered Care Model (PCCM) in home care. Home Health Care Management and Practice.	USA
Doherty M, Thompson H. 2014. Enhancing person-centered care through the development of a therapeutic relationship. British Journal of Community Nursing.	Northern Ireland

### Research strategy and selection criteria

This integrative review took into consideration all available studies exploring the description or definition of client/patient-centered care in home health care. The literature search was conducted on the 4^th^ of May 2020 using the electronic databases Medline, Scopus, Social Service Abstracts and Web of Science electronic databases. The search strategies used with the databases are presented in Appendix A and B. The second phase of the search process was conducted manually based on reference lists compiled from all eligible articles.

The correspondence author and an information specialist planned the search strategy. The information specialist and two researchers verified information retrieval independently. All studies concerning home care for older people aged over 65 and above using qualitative, quantitative and mixed methodologies were included in the review.

The following pre-agreed inclusion criteria were used in the selection process: a study concerning home care of older people aged 65, reporting the results of an empirical study or systematic review, has been peer reviewed, and full text is available. The information retrieval resulted in 742 articles. One of the selected articles dealt with medical home care involving the provision clinical treatment. Two additional articles were selected from material not included in the search results. Original articles were selected on the basis of their titles, summaries and full text.

**Figure 1.**
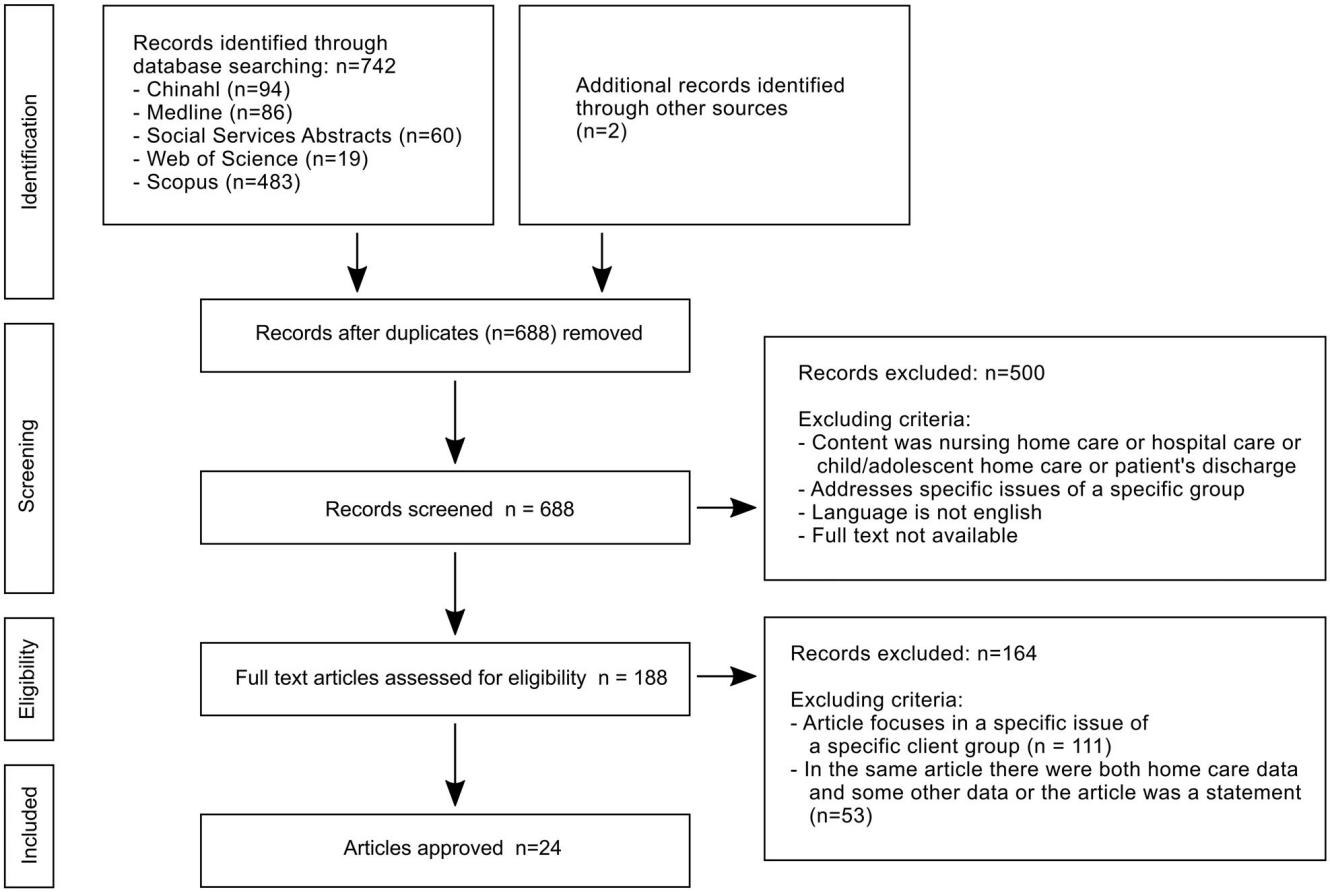
PRISMA flow diagram [22].

Exclusion criteria were as follows: the study was concerned with care provided in a nursing home or home care for children or adolescents, patient discharge, adolescence or specific issues of a specific group, the article had been published in a language other than English, or full text was not available (Figure 1).

### PRISMA screening and quality appraisal

This integrative review utilized the Preferred Reporting Items for Systematic Review and Meta-Analysis (PRISMA; Figure 1) [22].

The data search identified 742 articles, and additional searches of other sources identified further 2 articles. Firstly, the topics of the studies were assessed and duplicates removed. Subsequently, the remining articles (*n* = 688) were screened by title and abstract for relevance, as a result of which non-relevant articles (*n* = 504) were excluded. Full texts (*n* = 188) were screened for eligibility based on the inclusion and exclusion criteria. After the PRISMA screening process, 24 studies were included in the review. Two researchers assessed the full texts and 24 were included for final analysis by consensus. The selected articles are described in Tables 1–4.

The quality appraisal of the selected articles was carried out independently by the second reviewer and consensus was consequently reached. The quality assessment of the original studies (*n* = 24) selected for the review was carried out by two researchers using the evaluation criteria of the Joanna Briggs Institute [24] and the double-blind method. The following evaluation lists defined by the Joanna Briggs Institute were used in the evaluation:

The JBI Critical Appraisal Checklist for Analytical Cross Sectional Studies (7 articles), JBI Critical Appraisal Checklist for Quasi-Experimental Studies (non-randomized experimental studies; 1 article), JBI Critical Appraisal for Care Reports (1 article), JBI Critical Appraisal Checklist for Systematic Review and Research Synthesis (7 articles), JBI Critical Appraisal Checklist for Case Reports (1 article), JBI Critical Appraisal Checklist for qualitative Research (5 articles) and JBI Critical Appraisal Checklist for Text, Opinion papers (1 article) and JBI Critical Appraisal Checklist for Studies Reporting Prevalence Data (1 article).

The scales have between 6 and 11 questions with the response alternatives yes, no, unclear or not applicable. Affirmative responses for at least half of the questions were required in order to select an original article for the review [24]. The articles selected to the data set met the quality evaluation criteria (Appendix C–G).

### Data extraction and analysis

The data synthesis was implemented following the integrative review methodology by Whittemore and Knafl (2005). Details of the methods and outcomes organized, coded, categorized, and summarized based on their relevance to client-centered care were extracted from the primary sources. Thematical analysis was implemented by the correspondent author in data analysis by focusing on expressions of client-centeredness using the line-by-line analysis method [22,25]. Expressions were tabulated and coded. The list of codes was grouped into sub-themes and turned into categories with names characterizing their content. A concept map was generated from the relevant data. Analytical themes were defined and related to the outcomes of client centered care and the theoretical framework. After data comparison, concepts similar to one another were regrouped, condensed and refined. Finally, the concepts were contextualized based on the authors’ professional preconceptions as nursing teachers and researchers of health and social services. The selected studies were evaluated by four authors to assess the levels of credibility. All the remaining selected studies were found to be acceptable [21,23].

## Results

### Study characteristics

The original studies selected for the review had been published between 2007 and 2020 (Table 1). The studies had been carried out in Sweden (*n* = 5), USA (*n* = 3), Norway (*n* = 5), Canada (*n* = 3), Netherlands (*n* = 1), Belgium (*n* = 1), New Zealand (*n* = 1), Faroe Islands (*n* = 1), UK (*n* = 1), Spain [1], Northern Ireland (*n* = 1) and Finland (*n* = 1). The number of quantitative studies was 10 [1–10], qualitative studies 5 [11–14,26], literature reviews 7 [15–21] and case studies 2 [22,23].

### Elements of client-centered care in home care

As a result of thematical analysis, the client-centered care in older people home care was structured according to four analytical themes; clients’ involvement, the participation of family members and care partners, communication and collaboration, and evidence-based service competence.

#### Clients’ involvement

In this review, the client’s involvement has been defined as self-care, decision making and satisfactory daily life. Clients’ involvement in their own care is at the core of client-centered home care [27–29].

*Self-care.* In home care, motivation and commitment to one’s own care strengthens the individual performance and resources of the older person [29–33]. Participation in care management increases the sense of involvement and wellbeing [29,34–37]. Motivation is influenced by personality [38], cultural background [39], cognitive abilities, age and resources to participate, marital status and positive guidance [35]. The client’s knowledge, need of services, values, beliefs, and cultural background are incorporated into the planning and delivery of care [33,37,39]. Active involvement in the setting of goals is evidently one of the essential steps towards empowering older people [24,29,30,35,37,38]. Participation in medication, care planning and scheduling, and goal setting increases the client’s sense of involvement [30–32,35–37]. Involvement increases the clients’ satisfaction with care. Clients have a negative attitude towards their possibilities for influencing their own care, in contexts such as scheduling home visits and the continuity of care [40–42].

*Decision-making.* Influence over decision-making regarding personal care goals, an individual care plan, and relationships with professionals increase the client’s health, quality of life and satisfaction with care [28,31,35,37,40]. A lack of participation in decisions is in contradiction with the clients’ psychological need to have an influence over decisions affecting their own lives [27,28,33,35,43]. Client involvement with care management helps older persons obtain decisions that are meaningful for them and which make the persons’ life worth living [28,34,36,43]. It also increases dignity and autonomy of the older person [33,39].

*Satisfactory daily life.* Paying attention to the clients’ emotional and psychological needs promotes mental wellbeing and a sense of involvement in their own life at home [34,40–42]. Clients need a meaningful daily life and their existential needs must been taken into account. There are no significant differences between men and women in this regard [33,44]. It is important that the older persons’ home and living environment enable them to live actively, and maintain social relationships and interactions, and that they are comfortable with their living environment [28,29,35,37,44]. Feeling safe, and living an inclusive and meaningful life is important at home, as this prevents the feeling of illness and, as a consequence, reduces unnecessary use of health services [32,35–37,45]. Social relationships and consideration of cultural and ethnic perspectives in care and service make the home care client’s everyday life meaningful. The support provided by an active life, social relationships and discussions can help older persons participate in social activities [28,33,35,37,40].

#### Family members’ and care partners’ participation

The analytical theme of family members’ and care partners’ participation is closely linked to the core of client care and is structured according to the descriptive themes of family and care partners’ commitment to care, and family and care partners’ competence of care.

*Family members’ and care partners’ commitment to care*. Family members’ level of satisfaction, trust and emotions influence their commitment to an older person’s care [35,37,46,47]. They need to be provided with a possibility to assess their own needs, burdens, experiences, hopes and fears. Often, family members feel that they are not getting the psychological support they need to commit to the care process [24,28,44,46]. Families often experience pain or difficult emotions related to their family member’s health or increasing need of treatment [24,27,28,32,44]. The opinions, values, beliefs, knowledge, cultural background and feelings of family members should be respected to ensure their commitment to treatment [33,39]. The older person’s health and goals of care are defined in partnership. Shared decision-making in the care process is important between the client, family members and home care professionals. Client-centered care represents a service feature which is a significant contributing factor to family members’ commitment to care [27,30,32,46].

*Family members’ and care partners’ competence in care.* Family members and care partners are often part of a therapeutic alliance, participate in the implementation of the client’s care, and allow a better response to critical symptoms and warning signs. Family members and care partners are familiar with the older person’s health situation and life circumstances, they ensure that decisions respect the older person’s wants, needs, preferences, and ensure the education and support of the client [30,31,46]. Thus, it is important to recognize their knowledge base of care, lifestyle and the communication pattern in the family. In client-centered care, collaboration, the therapeutic relationship, and team spirit, flexibility and negotiation need to be realized between client, family members, professionals and home care service providers [29,32,36,40,43,45].

#### Communication and collaboration

The analytical theme of communication and collaboration is structured around the following descriptive themes: communication models, empowerment and partnership.

*Communication models.* Clients and their family receive timely, complete, and accurate information to communicate effectively [27,39,42,46]. In the communication models of client-centered care, the following items were emphasized: active listening, recognition of content, active questioning and prompting, interpretation of tips and cues, handling unclear reactions and learning to apologize, respect and understand the client’s life. More attention needs to be paid to the client’s perspective and views [37,41–43]. The communication skills, empathy, mindfulness, showing interest in the client, and emotional intelligence and self-knowledge are significant characteristics of professionals [29,37,41,43,44,48]. Humor and a friendly approach can be used as a strategy to support older people’s connection to everyday life as well as a strategy in handling the challenges pertaining to continuity and predictability. During home visits, humor was adapted to the home care context while at the same time used with sufficient sensitivity when interacting with the infirm older person and her everyday life [31,33,37,43].

*Empowerment.* Professionals who make room for and listen to the client enable their clients to deal with their own emotions. When professionals ask their clients about care, clients will be open about their emotional needs. Mutual non-verbal communication differs from that occurring in a hospital. Silently listening to the patient or client can promote building a connection with the patient [24,32,42,45,46]. Knowing the client comprehensively, openness, sensitivity, sense of humor, understanding, empathy, emotional intelligence, supportive space and trust are significant parts of interaction. Sensitivity also involves understanding and processing negative emotions as well as knowing, which topic to avoid [33,41,43,45,46,48].

Clients show their feelings and especially their negative emotions as clues. The nurse should respond to and make room for these feelings in positive encounters [41,42,47]. Organization of services can negatively influence the fulfilment of emotional and psychological needs, especially if there are differences between the continuity of care and the client’s hopes and needs for the future [24,30,34,47].

*Partnership.* The client-centered approach involves designing, implementing and evaluating client care based on mutual partnerships in collaboration with family members. Clarity when sharing information [27,28,33,43,45,47] facilitates collaboration. Listening to clients and encouraging them to explain about their lives, making genuine efforts to obtain feedback, and suggesting actions as responses to health changes contribute to building a relationship, and show compassion for older people’s concerns [32,33,48]. The decision-making process is simplified when there is a clear statement of what is possible and what is not [27,30,32,42,43,46]. To achieve this kind of communication, equality and co-operation, the family members involved in care provision may need training, information sources, support, encouragement and compassionate attitudes displayed in a variety of ways [24,30,31,43,46].

#### Evidence-based service competence

The analytical theme of home care service competence was structured as the delivery and organization of services, versatile clinical skills, implementation of services, and quality and safety outcomes.

*Delivery and organization of services.* In the delivery and organization of services [27,30–34,37,39,45] client-centered services are implemented in a highly coordinated, predictable, accessible, flexible and multidisciplinary manner in the provision of social and health care. Resource allocation and support within services also affect the organization of client-centered services, for example the need for small care teams [34,37,46,48], involving f a physician, geriatrician or a general practitioner in the provision of services, use of care technology in the delivery of services as well as the involvement of private and public sectors [39].

*Implementation of services.* In the implementation of services [24,31,32,34,35,37,39,40,44–46,48], client-centered care is related to the planning of care, scheduling of home visits, monitoring of care performance and outcomes, support of self-management and knowledge, effective professional communication, sensitivity, healthy culture and an evidence-based knowledge base. Identification of goals, ethics of care, task-orientation and continuity of care are important elements of implementation. Nurses have to achieve a balance between the fulfillment of the client’s needs and demands of organizations and professional standards [49].

*Versatile clinical skills.* Home care documentation is mostly concerned with medication. Home care professionals do not deal with issues more serious than respiratory problems, follow-up treatment, life cycle and health behaviors. The treatment they provide is primarily focused on the clients’ physical needs, whereas the clients’ other needs are not taken into account. The care and services are not based on the client’s individual life history and health status [40].

Significant client-centered clinical skills include the monitoring of the effects of medications, individual care design, clinical decision-support, communication skills, chronic care management and medication-related knowledge, documentation and disease management [24,28,29,39–41,45].

Client-centered service requires service needs assessment, development of clinical expertise, communication skills, empathy and interpretation of the client’s symptoms and signs. Client-centered work also requires changes in the working culture and reorganization of work shifts with different roles and specific ethical questions. Client-centered work increases the sense of staff capability and work satisfaction, work efficiency and commitment to work [28,35,36,39,45,47].

*Quality outcomes and personnel wellbeing.* The quality of home care services is achieved thorough the relationship between client, family members and professionals. Client-centered care can be a way to improve the quality of care, save costs and increase care satisfaction. Ideally, medical and care services for older persons should be better integrated in order to improve the availability of the services offered, the coordination of care and communication between providers in different service domains [29,32,37]. A lack of continuity and predictability poses a challenge and causes concern to the older person. Nurses’ visit schedules have also emerged as a major problem for home care clients [33,49].

The complexity of patient care and the need for co-operation and joint decision-making mean that there is a need for a focus on personal care and practical improvement of the patient experience [38,39]. Restructuring of improved service quality requires systematic political decision-making [27,32,49]. From the viewpoint of home care professionals, client-centered care and service increases the job satisfaction of personnel and create new roles for nurses [28,38,49].

## Discussion

The practical approach involves creating and promoting client-centeredness and therapeutic relationships between patients, nurses and other important people in their lives. It is supported by respect for individual persons’ values, right to self-determination, mutual respect and understanding [9,13,15,40,44].

The concept ‘client’ is related to caregivers and nurses in the home care context. The concept aims at improving the client’s health instead of being merely used for describing the context. It is realized at home, outside of the hospital. Home care clients need a wide range of help in their life situations. The concept is different from ‘patient’ which is functional and concentrates primarily on treating an illness [9,10,12,13,50,51].

(1) The category *Client involvement* describes the possibilities participate in self-care and the management of one’s own care process. The importance of shared decision-making has been described in several studies [13,20,26]. In the studies presented here, shared decision-making appears in the form of negotiations. This process influences commitment to care, functional capacity of self-care and satisfactory daily life. The client’s involvement seems to lie at the core of a successful home care process [6–8,20,40].

(2) The category *Family members’ and care partners’ participation* describes the therapeutic alliance of home care as well as the conditions and opportunities for participation in care [8]. In the past, several studies have noted that family members find it difficult in many ways to participate in the care process [11]. In the future, more attention should be paid to the competence, involvement and wellbeing of family members and care partners. From a family perspective, client-centered care is a partnership that takes into account capabilities, knowledge, opportunities and the feelings of family members [16].

(3) The category *communication and collaboration* describes the significance and individuality of interaction in home care. The use of communication should be purposeful, taking into account context, the goals of care and the various roles of nurses [13,50,51]. Communication has to be positive, involve giving emotional support and bearing in mind special characteristics of an old person in communication and in the interpretation of messages. Home care organizations should learn more about communicating with the clients [41].

Nurses need to be flexible in their different roles. Communication is at the core of achieving care goals, the objectives are to empower the client, family and care partners; and to create and strengthen partnerships [46]. Engagement in communication is also an important part of creating a sense of security in the client [13–15,17,50,52].

(4) The category of *evidence-based service competence* describes organizations and factors that underlie client-centeredness as well as the positive consequences and outcomes of client centered-care. In addition, client-centeredness is directly affected by political decision-making and funding models, service delivery and coordination, and the implementation planning and culture of organizations [38,52,53].

## Limitations

Only English language sources were used. In addition, the results might have been influenced by differences in the meanings of concepts used in social and health care.

## Conclusion

This review compiled research knowledge regarding client-centered care and its underlying factors. Client-centeredness in home care demands the client’s genuine involvement in self-care and decision-making in collaboration with family members and care partners. Communication is at the core of the care relationship, and competence development is essential given the context of home care and the individuality of the older person. In practice, evidence-based service organization expertise requires a significant change in service systems, organizational cultures and financial systems.

## Appendix A. Search strategies in the databases medline and cinahl, social service abstracts, scopus and web of science.

**Table:**

Search strategy in Medline and Cinahl	
S17	home care AND evaluation AND elderly
S16	S6 AND S10 AND S15
S15	S12 OR S13 OR S14
S14	AB aged or AB elderly OR AB "older person" OR AB "old aged" OR AB gerontologic* OR AB geriat*
S13	TI aged or TI elderly OR TI "older person" OR TI "old aged" OR TI gerontologic* OR TI geriat*
S12	(MH “aged/ elderly/old* person/old age/ gerontologic)
S11	S6 AND S10
S10	S7 OR S8 OR S9
S9	AB "patient focused care" OR AB "client cent#red care" OR AB "person cent#red care" OR AB "consumer cent#red care" OR AB "patient focused care" OR AB "person focused care" OR AB "consumer focused care" OR AB "family focused care"
S8	TI “patient focused care” OR TI “client cent#red care” OR TI “client cent#red care” OR TI “person cent#red care” OR TI “consumer cent#red care” OR TI “patient focused care” OR TI “client focused care” OR TI “person focused care” OR TI “consumer focused care”
S7	(MH “Patient Centered Care”)
S6	S1 OR S2 OR S3 OR S4 OR S5
S5	AB "home based care" OR AB "domiliciary care" OR "district nursing" OR "home service"
S4	TI “home based care” OR TI domiliciary care”
S3	AB “home health service*” OR AB “home care”
S2	TI ”home health service*” OR TI “home care” OR “Home Health Care”
S1	(MH ”Home Health Care+”)

## Appendix B. Search strategies in the databases social service abstracts, scopus and web of science.

**Table:**

Database	Search strategy
Search strategy in Social Services Abstracts	(“home health service*” OR “home care” OR “district nursing” OR “home based “ OR “domiliciary care”) AND (“patient centered care” OR “client centered care”
Search strategy in Scopus	TITLE-ABS-KEY ((client-centered AND care OR person-centered AND care OR patient-centered AND care) AND (home AND care AND service* OR home AND health AND care OR district AND nursing) AND (aged OR eld* OR senior* OR ger*))
Search strategy in Web of Science	((“client-centered care” or ”person-centered care” or “patient centered care”) AND (“home care service*” or “home health care” or “district nursing”) AND (aged or eld* or old* or senior* or ger*))

## Appendix C. Quality assessment with JBI critical appraisal checklist for qualitative research.

**Table:**

JBI Critical Appraisal Checklist for Qualitative Research	Roin 2017	Kristensen et al. 2017	Öreland et al. 2008	Kuluski et al. 2019	Sundler et al.,2019
1. Is there congruity between the stated philosophical perspective and the research methodology?	y	y	y	y	y
2. Is there congruity between the research methodology and the research question or objectives?	y	y	y	y	y
3. Is there congruity between the research methodology and the methods used to collect data?	y	y	y	y	y
4. Is there congruity between the research methodology and the representation and analysis of data?	y	y	y	y	y
5. Is there congruity between the research methodology and the interpretation of results?	y	y	y	y	y
6. Is there a statement locating the researcher culturally or theoretically?	y	y	y	y	y
7. Is the influence of the researcher on the research, and vice versa, addressed?	y	y	u	n	y
8. Are participants, and their voices, adequately represented?	y	y	y	y	y
9. Is the research ethical according to current criteria or, for recent studies, and is there evidence of ethical approval by an appropriate body?	y	y	y	y	y
10. Do the conclusions drawn in the research report flow from the analysis, or interpretation, of the data	y	y	y	y	y
Total	10	10	9	9	10

Note Key: Y=Yes, N=No, U=Unclear, NA= Not applicable.

## Appendix D. Quality assessment with JBI critical appraisal checklist for systematic reviews and research synthesis.

**Table:**

JBI Critical Appraisal Checklist for Systematic Reviews and Research Synthesis	Ruggiano N and Edvardsson D 2013	Wilberforce et al. 2016	DePuccio M and Hoff T 2014	Anker-Hansen et al. 2019	Carvajal et al. 2019	Giosa et al. 2019	Olsen et al. 2019
1. Is the review question clearly and explicitly stated?	Y	Y	y	y	y	y	y
2. Were the inclusion criteria appropriate for the review question?	Y	Y	y	y	y	y	y
3. Was the search strategy appropriate?	Y	Y	y	y	y	y	y
4. Were the sources and resources used to search for studies adequate?	Y	Y	y	y	y	y	y
5. Were the criteria for appraising studies appropriate?	Y	Y	y	y	y	y	y
6. Was critical appraisal conducted by two or more reviewers independently?	Y	Y	y	n	y	y	y
7. Were there methods to minimize errors in data extraction?	Y	Y	y	u	y	y	y
8. Were the methods used to combine studies appropriate?	Y	Y	y	y	y	y	y
9. Was the likelihood of publication bias assessed?	Y	Y	y	y	y	y	y
10. Were recommendations for policy and/or practice supported by the reported data?	Y	Y	y	y	y	y	y
11. Were the specific directives for new research appropriate?	Y	y	y	y	y	y	y
Total	11	11	11	9	11	11	11

Note Key: Y=Yes, N=No, U=Unclear, NA= Not applicable.

**Table:**

JBI Critical Appraisal Checklist for Case Reports	Silver et al. 2011	Doherty M and Thompson H 2014
1. Were patient’s demographic characteristics clearly described?	Y	Y
2. Was the patient’s history clearly described and presented as a timeline?	Y	U
3. Was the current clinical condition of the patient on presentation clearly described?	Y	Y
4. Were diagnostic tests or assessment methods and the results clearly described?	U	Y
5. Was the intervention(s) or treatment procedure(s) clearly described?	Y	Y
6. Was the post-intervention clinical condition clearly described?	Y	
7. Were adverse events (harms) or unanticipated events identified and described?	U	Y
8. Does the case report provide takeaway lessons?	NA	Y
Total	5	6

Note Key: Y=Yes, N=No, U=Unclear, NA= Not applicable.

## Appendix F. Quality assessment with JBI critical appraisal checklist for analytical cross sectoral studies.

**Table:**

JBI Critical Appraisal Checklist for Analytical Cross Sectoral Studies	Hafskjold et al. 2015	Brazil et al. 2012	Bosman et al. 2008	Sundler et al. 2017	Höglander et al. 2017	Van Eenoo et al. 2018	Turjamaa et al. 2015	Hafskjold et al. 2018
1. Were the criteria for inclusion in the sample clearly defined?	Y	Y	Y	Y	Y	Y	Y	y
2. Were the study subjects and the setting described in detail?	Y	Y	Y	Y	Y	Y	Y	y
3. Was the exposure measured in a valid and reliable way?	Y	Y	Y	Y	Y	Y	Y	y
4. Were objective, standard criteria used for measurement of the condition?	Y	Y	Y	Y	Y	Y	Y	y
5. Were confounding factors identified?	Y	Y	Y	Y	NA	Y	Y	y
6. Were strategies to deal with confounding factors stated?	Y	Y	Y	U	U	Y	Y	y
7. Were the outcomes measured in a valid and reliable way?	Y	Y	Y	Y	Y	Y	Y	y
8. Was appropriate statistical analysis used?	Y	Y	Y	Y	Y	Y	Y	y
Total	8	8	8	7	6	8	8	8

Note Key: Y=Yes, N=No, U=Unclear, NA= Not applicable.

## Appendix G. Quality assessment with JBI critical appraisal checklist for Quasi-Experimental studies.

**Table:**

JBI Critical Appraisal Checklist for Quasi-Experimental Studies	Bölenius et al. 2017	Parssons and Parssons 2012
1. Is it clear in the study what is the ‘cause’ and what is the ‘effect’ (i.e. there is no confusion about which variable comes first)?	Y	Y
2. Were the participants included in any comparisons similar?	Y	Y
3. Were the participants included in any comparisons receiving similar treatment/care, other than the exposure or intervention of interest?	Y	Y
4. Was there a control group?	Y	Y
5. Were there multiple measurements of the outcome both pre and post the intervention/exposure?	Y	Y
6. Was follow up complete and if not, were differences between groups in terms of their follow up adequately described and analyzed?	Y	Y
7. Were the outcomes of participants included in any comparisons measured in the same way?	Y	U
8. Were outcomes measured in a reliable way?	Y	Y
9. Was appropriate statistical analysis used?	Y	Y
Total	9	8

Note Key: Y=Yes, N=No, U=Unclear, NA= Not applicable.
